# 

**Published:** 1906-07

**Authors:** 


### INDEX.

Abstracts and Selections......34, 80, 112, 157, 206, 242, 278, 331, 355, 399, 437........................................ 484
A Case Presenting Many Interesting Features. By R. E. B. Bledsoe, M. D., Somerville, Texas...................... 260
Adrenalin Chloride in the Treatment of Chronic Nasal, Post-
A Minor Surgical Operation for the Prevention of Conception, and Its Applications. By J. W. Kenney, M. D., San Antonio ............................................ 98
Nasal and Pharyngeal Catarrh. By John C. Warbrick, M. D., Chicago.................................... 184
Angina Pectoris. By Wm. A. Haley, M. D., Houston, Texas. 427
A Parody. By Fritz Lanham, Austin, Texas............. 145
A State Sanatorium for Consumptives.................. 269
A Theoretical "Cure" for Cancer........................ 107

Bon Bons.............................................. 24
Books and Magazines...84, 121, 160, 212, 281, 368, 408, 454, 494

Castration for Rape.................................... 393
Cause and Effect....................................... 475
Censorship of the Medical Press........................ 431
Clinical Lecture, Chronic Eczema. By John V. Shoemaker, M. D., LL. D., Philadelphia, Pa..................... 257
Complications of Gonorrhea and Treatment. By E. B. Parsons, M. D., Palestine, Texas....................... 191
Correspondence ................................151, 202
Criminal Responsibility of the Insane. By F. E. Daniel, M. D., Austin, Texas................................ 49

Difficult Diagnosis of Yellow Fever Cases. By L. Sexton, M. D., New Orleans, La........................... 10

Editorialets..........154, 204, 237, 274, 328, 349, 395, 435, 482
Emasculation for Criminal Assaults and for Incest......... 347

Federal vs. State Quarantine........................... 267
Fracture of the Ulna During Massage Following Operation for Colles' Fracture................................ 303

George R. Tabor, M. D., State Health Officer and Surgeon General of Texas. By the Editor...................... 1
Gunshot Wound of the Vertebra. By H. A. Moseley, M. D., Dallas, Texas .......................................... 104

History of Medical Teaching in Texas. By J. F. Y. Paine, M. D., Galveston, Texas.............................. 173
Hemorrhoids. By R. W. Skipper, M. D., Lovelady, Texas.. 186

Lagniappe ........................................... 77
Laryngeal Diphtheria, With Special Reference to Intubation. By T. J. Dodson, M. D., Mangum, Okla................ 337

Miscellany ........................................... 109
Management of Typhoid Fever. By Edward C. Register, M. D., Charlotte, N. C............................ 308
Methods of Anesthesia, Old and New. By W. F. Waugh, M. D., Chicago .......................................... 341
Mosquito Inhibition. By W. C. Abbott, M. D., Chicago, Ill.. 468

Notice to Delegates.................................... 148
Nursing ............................................. 347

Our Anniversary ...................................... 17
Odontalgia. By Price Cheaney, B. S., M. D., D. D. S., Dallas, Texas............................................ 297
On the Proper Selection of a Site for a Tuberculosis Sanatorium in the State of Texas. By C. H. Wilkinson, M. D., Galveston, Texas.................................... 383

Prostatic Enlargement With Some of the Causes and Complications. By W. R. Kone, M. D., Oklahoma City, Okla.. 463
Publisher's Department......44, 92, 126, 164, 212, 248, 288, 334, 372, 416.......................................... 460

Resourcefulness of Texas Doctors. Letter from Dr. Hughes.. 100
Rest as a Therapeutic Agent. By L. M. Barnes, M. D., Thorndale, Texas ...................................... 143

Some Remarkable Cases that I Have Seen. By W. A. Bedford, M. D., Franklin, Texas........................... 93
Shall the Profession Do Contract Work Cheaper Than for the Public? By J. P. Oliver, M. D., Caldwell, Texas........ 138
State Insane Asylum, Austin............................ 149
Society Notes.......................................... 240

Texas and the Great Tuberculosis Congress.................. 74
Texas and the New Federal Quarantine Law............... 75
Texas' New Health Officer and Surgeon General............ 270
The American International Congress on Tuberculosis....21, 73
The Duty of the Government to Its People. By F. E. Daniel, M. D., Austin, Texas.................................. 215
The Family Physician. By D. Monroe, M. D., Cameron, Texas 263
The General Practitioner and the Railroads. By P. W. Beckman, M. D., Beaumont, Texas.......................... 101
The Handwriting Is On the Wall. Imperial Methods in Medical Politics .......................................... 18
The Importance of Some Symptoms of Uterine Fibroids, with Report of Cases. By F. L. Barnes, M. D., Trinity, Texas .............................................. 7
The Medical Officers of the State......................... 147
The Mixed Board Bill.................................... 392
The New Medical Practice Act........................... 348
The New Medical Practice Act—The Mixed Board Bill..... 433
The New One Board Bill Medical Examiners for Texas, as Amended and Passed April 5, 1907.................... 377
The New One Board Bill Medical Examiners for Texas, as Amended and Passed April 5, 1907. (Effective July 17, 1907) ............................................ 419
The Pharmacopeia—As Seen by One General Practitioner. By J. Leverett, A. B., M. D., Yonkers, N. Y............ 425
The Pure Food Bill...................................... 19
The Recent Meeting of the American International Congress on Tuberculosis in New York......................... 233
The Scheme to Level All Distinctions in Medicine, Voiced by Senator Looney—Minority Rule—The Tail Wags the Dog.. 389
The Schoolroom as a Factor in Diseases of Young Girls...... 70
The Therapeutics of Laughter............................ 321
The Tuberculosis Congress............................... 197
The Use and Abuse of the Stomach Tube. By J. W. Torbett, M. D., Marlin, Texas................................ 3
The Value of the Reflex Arc in Diagnosis. by Marc Ray Hughes, M. D., St. Louis, Mo......................... 137
Therapeutic Tips. By E. S. McKee, M. D., Cincinnati, O.... 470
Tuberculosis and Its Prophylaxis. By O. I. Halbert, M. D., Waco, Texas ......................................... 131

"Unifying the Profession" in the Interest of the Great Medical Journal Trust—Proselyting the Pathies.............. 345

